# More than Mental Illness: Experiences of Associating with Stigma of Mental Illness for Chinese College Students

**DOI:** 10.3390/ijerph19020864

**Published:** 2022-01-13

**Authors:** Miao Yu, Shengli Cheng, Kenneth Po-Lun Fung, Josephine Pui-Hing Wong, Cunxian Jia

**Affiliations:** 1 Department of Social Work, School of Philosophy and Social Development, Shandong University, Jinan 250100, China; 2 Department of Psychiatry, Faculty of Medicine, University of Toronto, Toronto, ON M5S 1A1, Canada; ken.fung@uhn.ca; 3 Daphne Cockwell School of Nursing, Ryerson University, Toronto, ON M5B 2K3, Canada; jph.wong@ryerson.ca; 4 Department of Epidemiology, School of Public Health, Cheeloo College of Medicine, Shandong University, Jinan 250012, China; jiacunxian@sdu.edu.cn

**Keywords:** mental illness, stigma, Chinese college students, implementation study, focus group

## Abstract

From existing empirical research, we identified that Chinese college students commonly experience stigma surrounding mental illness and found some factors that support them in resisting the stigma and achieving psychological health. However, less research provides qualitative data involving individual experiences and insights on mental illness within this group of college students. This study, based on Linking Hearts (an internationally cooperative research-sharing project between China and Canada), was conducted in Shandong, Jinan, and aims to promote the mental health of college students by empowering interdisciplinary professionals and students. Through the research project, this study analyzed the materials from 24 focus groups, explored the understanding of mental illness and prevalence of mental illness stigma in Chinese colleges at the present time, administered a background questionnaire, and provided statistical support for some revealed themes. The final themes are as follows: mental illness is stereotyped as “severe, pathetic, and complicated”; the misconception of “visiting a psychological counselor is scary”; from public stigma to self-stigma; barriers deterring students from seeking help or accessing services; two sides of the same coin: peer support versus peer pressure.

## 1. Introduction 

Mental health problems among post-secondary students are becoming a priority issue in China as this population has experienced massive growth over the last two decades. In 2019, there were approximately 40 million college students at Chinese universities [1]. The number of college students suffering from mental illness has been increasing over time, and students’ mental wellbeing appears to be worse than the general population’s [2,3]. According to a previous national survey of about 126,000 college students, 20.3% appeared to have a mental health issue, including depression, anxiety, obsessive-compulsive disorder, poor interpersonal relationships, character issues, and other mental illnesses [4]. Some studies presented an even worse picture, in which almost 30% of college students were suffering from some kind of psychological barrier or illness—a much higher percentage than other young people of the same age [5].

As a response to these mental health needs, since 2005, the Chinese government has mandated the provision of at least two mental health counselors for every university, with the proportion of counselors to students being no greater than 1:4000, and that mental health education must be integrated into each college’s teaching program. In the *Outline of a National Plan for Education Reform and Development 2010–2020*, the Chinese government called for strengthening of mental health education [6], based on the report *Opinion on Further Strengthening and Improving College Students’ Mental Health Education* issued jointly by the Ministry of Education, the Ministry of Public Health, and the Communist Youth League. In 2016, the Chinese authorities published *Guidelines on Strengthening Mental Health Services*, the first comprehensive policy for strengthening mental health at the state level. Improving the mental resilience of college students was one of its most important goals, as was developing psychological crisis intervention.

Stigma against mental illness makes its treatment and recovery especially challenging, and this needs to be understood in its sociocultural context [7]. Corrigan [8] emphasized that the stigma of mental illness is embedded in personal experiences; it is in the worlds of stories, down to the individual person and individual events, that stigma as personally experienced can have a profound impact for a person with mental illness. However, mental illness stigma is not just an individual problem for people with mental illness. Stigma and discrimination arise from a social context and are of concern for all of society. Students who experience mental illness often do not seek professional help for several reasons: economic limitations, inadequate knowledge of support services, and fear of being stigmatized [9]. The WHO and World Psychiatric Association have recognized public stigma as the critical barrier to appropriate care and inclusion of individuals with mental illness in society. 

Stigma has a significant impact on the lives of individuals with mental illness. Existing studies have found that only 18% to 34% of young people with high levels of depression or anxiety seek professional help [10]. Many individuals who need to seek help for mental illnesses are not seeking services. This includes college students, who are at the most vulnerable age for risk of mental health issues such as depression and anxiety [11]. The stigma surrounding mental illness impacts how people access these services and constructs a barrier to the appropriate treatment and support. Moreover, the stigma surrounding mental illness is not just a problem for people with these disorders or for their families, nor is it merely a harsh reality of the world which we must all learn to overcome. 

This paper reports on the stigma of mental illness among Chinese college students. It is based on data collected from the first phase of Linking Hearts, an implementation science project between China and Canada. The aim of Linking Hearts is to promote the mental health of college students in Jinan, Shandong through capacity-building among mental health professionals and students. 

## 2. Literature Review

Stigmatization of mental illness is prevalent across all societies. However, as Eastern cultures are considered to be “collectivist” in comparison to Western cultures, there is a perception that stigma against mental illness may be more prevalent in Chinese society. It has been proposed that Chinese culture attaches more significance to the collective representation of families, in which having a mentally ill relative is regarded as something that may bring shame to the family. Hence, many Chinese families have concealed their relatives’ mental illness in order to avoid stigma [8]. Stereotypes, prejudice, and discrimination are components of stigma formation, which is a useful model for explaining the development of stigmas around mental illness. *Stereotypes* refers to public and dominant attitudes toward specific groups of people. *Prejudice* occurs when a person endorses a negative stereotype about a group; it is an emotional reaction resulting from agreement with public and dominant attitudes. *Discrimination* refers to the behavior that results from prejudice and represents the negative ways people act when they endorse a negative stereotype [8,12,13]. *Stigma* means stereotypes that reflect negatively on a group. Existing research has uncovered three sets of stigmatizing beliefs about mental illness that are commonly endorsed by the public. These include (a) *fear and exclusion*, which argues that persons with severe mental illness are dangerous to other people and should be feared; (b) *authoritarianism*, which claims that persons with severe mental illness are irresponsible; and (c) *benevolence*, which argues that persons with severe mental illness are childlike and need to be specially cared for [14]. To fully provide a basis for understanding stigma, specific stereotypes and prejudice within the categories of *dangerousness*, *incompetence*, and *permanence* are also emphasized [13].

Stigma of mental illness can be divided into *public stigma* and *self-stigma*. Public stigma refers to prejudice endorsed by the general population, resulting in discrimination against people with mental illness. Self-stigma occurs when people living with mental illness internalize the stigma and experience diminished self-esteem and self-efficacy [15]. A study in 14 European countries examined the association between public stigma and self-stigma and found that individuals with mental illness living in countries with less public stigmatizing attitudes scored higher rates of help-seeking, reported better access to service and information, had lower rates of self-stigma, and felt more empowered [16]. As public stigma and self-stigma are intertwined, initiatives to reduce self-stigma must take a multilevel approach. 

In addition, mental illness stigma can also manifest as *structural stigma and courtesy stigma*. Structural stigma refers to exclusive policies of governmental and private institutions that restrict the opportunities of people with mental illness [17]. Courtesy stigma, or associative stigma, applies to friends, family, service providers, employers, or other individuals who appear connected to the stigmatized group [18,19]. It is important to note that people belonging to more than one socially disadvantaged group often experience multiple intersecting stigmas [20]. 

As for ways to reduce stigma, one study discovered three strategies that could be conducted to achieve this result. These are contact (members of the general public who are encouraged to interact with persons with mental illness will be less likely to stigmatize), education (members of society who know more about mental illness are less likely to endorse stigmatizing opinions about it), and protest (stigma may decrease when important segments of society clearly state that “These discriminatory actions are not acceptable”) [8]. A study with college students that examined the association between stigmatizing attitude and contact with people living with mental illness showed that having contact with persons with mental illness reduced stigmatizing attitudes toward them [21]. Similarly, a study about attitudes toward mental illness among university staff showed that individuals who had higher levels of education, personal experience with mental illness, or professional roles in mental health were more likely to demonstrate acceptance of people living with mental illness [22]. 

Other studies showed that mental-health-related help-seeking behaviors among students were influenced by perceived stigma and mistrust. Some students preferred to solve problems related to their mental health by themselves or with their friends and families, rather than by using professional counselors [23]. A study on the relationship between the stigma of mental illness and attitude towards psychological help revealed that there were significant differences according to participants’ gender, grade level, and history of psychological help. The results also indicated that eliminating stigma and encouraging students to seek psychological counselling is critical to supporting students to overcome mental health crises [24]. Other studies have shown that attitudes towards seeking psychological help are not merely individual choices, but decisions influenced by existing social supports [23,25]. While existing studies have provided important insights about stigma of mental illness among Chinese college students, most of these studies used quantitative methods. There is limited knowledge on the contexts and nuances of college students’ experiences and perspectives. This paper draws on the qualitative data of the Linking Hearts Study and contributes to narrowing the identified knowledge gap.

## 3. Methods 

### 3.1. The Linking Hearts Implementation Study

The Linking Hearts project is an implementation science research being undertaken by a multidisciplinary team of researchers from China and Canada. The aim of the project is to reduce mental illness stigma and promote the mental health of university students in Jinan, Shandong, China through the implementation of the Acceptance and Commitment to Empowerment (ACE) intervention. The project consists of three components: (1) *contextual assessment and analysis* with service providers and students to inform the adaptation of the Acceptance and Commitment to Empowerment (ACE) intervention; (2) *implementation* of the refined ACE intervention with service providers and university students; and (3) *integrated knowledge translation* to engage diverse groups of knowledge users throughout all stages of this study [26]. 

The purpose of the contextual assessment and analysis was to acquire a deep understanding of the perspectives and experiences of mental illness stigma among university students and mental health professionals. We used mixed methods of surveys and focus groups. As indicate above, most mental health studies of college students in China have drawn on quantitative surveys to identify causal relationships. Limited studies have used qualitative methods to explore mental illness stigma. Thus, we also used focus groups in data collection to acquire a comprehensive understanding of the contexts of the participants’ perspectives and experiences. The focus group method allows us to engage a number of individuals in a focused discussion simultaneously. It enables us to capture a range of ideas, observe participants’ emotions, and gain insights about the similarities and differences in participants’ perspectives [27]. One of the distinct features of the focus group method is the presence of group interactions and dynamics, which often reflect participants’ attitudes, values, and beliefs. Hence, the type and range of data generated through the social interaction in focus groups are often deeper and richer than what can be obtained from individual interviews or surveys [28]. 

### 3.2. Data Collection 

We used purposive sampling to recruit participants based on the aim of the study and the research questions [29]. We engaged two groups of participants: college students and mental health professionals, including psychological counselors, educators, and staff of student affairs. With the students, the sampling criteria including balanced gender, different grades (most from the senior grades which means more rich experiences in accessing mental health services), and different majors (both humanities and social science and science should be included), who are interested in mental health among college students. The interview focused on their understanding of mental illness and their experiences in accessing mental health services. With the professionals, the sampling criteria included in-service staff who are working with students, and different professions were preferred (psychological counselors, college instructors, medical staff from the school infirmary). The interview focused on their perspectives on the mental health needs of college students and suggestions of relevant mental health care. Between May and September in 2019 we conducted 12 focus groups with 144 mental health professionals (12 participants per group) and conducted 12 focus groups with 144 college students (also 12 participants per group). The focus group participants were recruited from the six collaborating universities: (1) Jinan University; (2) Shandong Women’s University; (3) Shandong University; (4) Shandong Youth University of Political Science; (5) Shandong Jianzhu University; (6) Shandong Normal University. Two groups of students and two groups of professionals were conducted at each university with 12 participants per group (12 × (2 + 2) × 6 = 288). These participants were recruited by both online and offline means which depended on the different universities; recruitment letters were posted online and the research leader and members from the six universities mobilized the principal for college instructors and colleagues around them who matched the sampling criteria, and the participants received RMB 200 after completed the focus group for incentive payments. Every focus group was conducted with two coordinators and one observer, all the participants completed the informed consent forms and background data collection, and these focus groups were conducted at each university. As we mentioned earlier, this study belongs to the Linking Hearts Implementation study; as the contextual assessment and analysis, both qualitative focus groups, and quantitative survey were conducted at this stage, there were also 600 background questionnaires (100 questionnaires each university) administrated. We only analyzed the focus groups in this study and the descriptive quantitative data presented as the supportive material for the related emerging qualitative theme.

### 3.3. Data Analysis

The focus groups were conducted in Mandarin and were audio-recorded with written consent from the participants. The interviews were transcribed verbatim in Chinese and then translated into English. In analyzing the focus group data, we applied framework analysis to identify explanatory patterns within and across focus groups. Krueger and Casey [30] emphasize the importance of using analytical strategies that are systematic, sequential, verifiable, and continuous to minimize any potential bias in analyzing and interpreting focus group data. In framework analysis (Ritchie and Spencer, 1994), coding is one of the key processes to categorize and classify responses from participants. Specifically, we engaged in five stages of analysis: (1) familiarization with the data; (2) identifying a thematic framework by writing memos and developing categories; (3) indexing and comparing quotes within and across cases or groups; (4) coding the quotes; and (5) mapping and interpretation of the data. Although framework analysis adapts a thematic approach, it allows themes to develop both from the research questions and from the narratives of the participants [27]. To protect the anonymity of the participants, the quotes we present in this paper will only identify participants by the category of participants, i.e., students or professional, and the case or focus groups they engaged in. For example, “1S2, p1” represent case 1 from the second students focus group from the first university, (1P2, p2) means the case 2 from the second service provider’s focus group from the first university.

## 4. Findings 

The questionnaire results revealed that mental health challenges were very common among the students: 80% reported anxiety, 80% reported video addiction problems, 64% reported obsessive-compulsive disorder, and 53% reported experiences of depression. In this paper, we report on the results of the focus groups, which have given rise to a number of key themes. These qualitive rich descriptions help to contextualize and enhance our understanding of the quantitative findings. 

### 4.1. Mental Illness Is Stereotyped as “Severe, Pathetic, and Complicated”

With the focus groups of college students, self-perception was a key factor, including self-conception, self-evaluation, and lack of clarity about their abilities. This poses challenges in term of career planning and job hunting: some felt inferior while others felt superior, some students did not know how to regulate their emotions, and so on. Almost all the research participants highlighted the importance of college students’ mental health, especially since these students are in the transitional period from adolescence to youth. In the focus groups, we invited the college student participants to share their feelings about mental illness. Some students defined severe conditions such as depression as mental illness. Others perceived a person living with mental illness as someone difficult to get along with.

*“Regarding mental illness, depression is the first shot for me, especially those people who killed themselves, with such severe depression that I couldn’t imagine”.*

(2S1, p6)

*“I know some of my students with mental illness. I realize they see things always in a bad way, a very negative way, and it seems hard for them to see things in a whole picture. Mental illness comes from themselves, in my opinion, if they could understand the world with a more positive way, they would be getting better”.*

(2S1, p12)

*“There is a big difference between mental illness and mental health problems. I believe the issue of mental health problems is quite common among us; however, mental illness is a kind of severe mental disease”.*

(3S1, p7)

Besides regarding mental illness as always severe, some of the student participants stated that people who have mental illness were a little pathetic: students believed either that something horrible had happened in their childhoods, or that they encountered great stress or difficulties in their daily lives. Meanwhile, some participants argued that mental illness was not opposite to mental health. It was very difficult for them to define it with a clear statement. They believed that in daily life, there was no determinative standard to distinguish mental health from mental illness, because all people experience depression or anxiety when facing the changing society of today.

*“Mental health and mental illness are both difficult to define clearly. I think everything has two faces like every coin has two sides; mental illness is a concept relative to mental health. No one could claim that he or she is in perfect mental health, nor could anyone be defined as totally mentally ill. Individuals can experience both mental health and mental illness: they emerge in different times for different situations. It presents discrimination if we define someone as totally mental illness as abnormal”.*

(2S2, p5, p9)

Besides participants’ understanding of the nature of mental illness, some responses expressed their perspectives on mental illness: regardless of how difficult participants found it to correctly define mental illness, they stated that mentally ill persons were objectively in a situation of exclusion, even admitting that in some situations they themselves would exclude individuals with mental illness.

### 4.2. The Misconception of “Visiting a Psychological Counselor Is Scary”

How students perceived psychological counselors also revealed their perspectives on mental illness. If students’ impressions and definitions of mental illness were influenced by negative stereotypes, their attitudes towards psychological counselors also revealed public attitudes about mental illness. Based on the responses of the participants, when they met mental difficulties, it seemed to be difficult for students to visit psychologists.

*“When talking about mental health, it always reminds me of the questionnaires about mental health sent by the university, and also the thought of vising a psychiatrist or a psychological counselor is scary”.*

(3S2, p2)

*“Regarding people with mental illness, in my opinion these people are patients who need to see doctors, which makes me uncomfortable. However, one of my classmates regularly sees the psychological consultant in the mental health center at our university. He changed my perspective; I realized it is not a big deal if you encounter some mental problem”.*

(5S2, p3)

Although they showed fear of psychological counselors, they trusted the counselors’ authority on mental health. Some of the participants would prefer to ask counselors rather than friends for help if they faced mental troubles.

*“The professional support gains trust from me rather than the friends or classmates around me; these professionals represent knowledge and authority in the psychological area”.*

(1S1, p1)

*“Because they have less knowledge about mental health, when we suggest that our students see a psychological counselor if they meet this kind of trouble, they are always terrified by the suggestion. They often treat this as very serious problem; they refuse to admit it consciously; even if they went to see the doctors, they were very worried about their privacy and hoped the doctors could keep their secrets from other people, and they always made sure of that with the doctors. From my experience, these students are afraid their mental illness will be discovered by others, which means acceptance and reduction of mental illness stigma are pretty important for our society”.*

(1P1, p9)

Some service providers who shared their experiences working with the parents of students with mental illness stated that parents rarely cooperate with the doctors, while also rejecting the mental illness of their children. The parents complain that “It’s too embarrassing if we bring our child into hospital.” However, mental illness stigma seemed not to be a problem with some parents. One participant (2P2, p11) noted that parents are well educated nowadays: they demonstrate tolerance of mental illness issues, and it seems acceptable to them if their child encounters mental problems. Besides the stigma issue, however, there is another barrier for students in accessing services or supports: many students have no chance to access professional services because of a financial problem (6P1, p5). There are some conflicts among the responses of service providers: some of them believed that mental illness has become more acceptable to the public, some said that parents and students showed a more positive attitude towards dealing with mental illness, and some said that parents and students showed an open mind to treating the illness. Meanwhile, they still emphasized that some parents continue hiding their children’s illness from other people, so it is possible that the stigma of mental illness is not reduced but has simply become more covert.

### 4.3. From Public Stigma to Self-Stigma: Barriers Deterring Students from Seeking Help or Accessing Services

Based on the self-report questionnaire, there were six top barriers for college students in achieving treatment and support, including fear of judgement/ discrimination from the community, privacy and confidentiality concerns, lack of safe spaces/privacy for services, access barriers related to service locations/opening hours/costs, and lack of school/ public policies to protect against mental illness discrimination. The specific percentages for these are presented in Table 1.

In the students’ focus groups, the participants responded that among the barriers deterring them from seeking help or accessing services, the stigma of mental illness was the essential concern. Some college students stated that the easiest way to obtain help is through the students’ own network—the counselling center was always the last place that students wanted to seek help from, because to them consulting professionals meant that they were officially seriously ill or even abnormal.

*“For the people who have mental illness, I would say that maybe I will leave them alone rather than help them. I would offer support if they asked for it. It’s dangerous to be around them; in my opinion, the safety of myself is the most important thing”.*

(1S1, p8)

*“The whole society pays not much attention to mental illness, even downplaying its bad influence. If some student did horrible things to himself, the usual response was to cover up the information. Maybe the school worried about the panic among the college students. However, making it public could encourage students earn to ask for help when he/she encountered a mental illness issue”.*

(1S1, p9)

Public stigma refers to a set of negative attitudes and beliefs that motivate individuals to fear, reject, and avoid people who suffer from mental illness. For example, persons with mental illness are more likely to experience employment discrimination and social relation exclusion [31]. Self-stigma is a multilevel process that begins with awareness of public stigma. A person with mental illness internalizes the stigma and applies it from people with mental illness in general to the individual [32]. According to the study parameters, the participants were college students and mental health service providers who were not experiencing mental illness themselves; what they described and interpreted were environmental factors or public attitudes towards mental illness, rather than subjective interpretations of their own mental illness or personal experiences. As stated earlier, the deployment of public stigma is mostly reflected in that mental illness was mainly defined as something severe and pathetic, or that visiting the psychological counselor was regarded as very horrible existence. However, there is still the factor of how the college students internalize negative public attitudes into self-cognition: 75% of the participants who completed the questionnaires felt fear of judgment and discrimination from their peers, as shown in Table 1.

### 4.4. Two Sides of the Same Coin: Peer Support versus Peer Pressure

Based on the discussions among focus groups, these college students are more easily influenced by their classmates and friends. On one hand, they admitted that students who were members of a “Committee on Psychology” could help them to some extent (“Committee on Psychology” is a program developed in some studied universities to promote peer support for college students’ mental health: some students are chosen to help their classmates who are experiencing mental problems). However, how to qualify these chosen students is a troubling issue.

*“We can develop the “Committee on Psychology” program, which encourages supervision from peers. These “mind guiders” need qualified training; they should be taken seriously and trained regularly rather than only having emphasized the interest and responsibility they should have. Because some mind guiders are really helpful, they could have a more positive influence if they get professional and strict education and training”.*

(1S1, p10)

The “Committee on Psychology” program is an innovative action plan for promoting and developing the mental health of Chinese college students. It has been conducted as an approach for promoting mental health since Tianjin University first developed this program in China in 2004. This approach is becoming a very important part of the mental health services for college students. According to the statistics presented in the “Blue Book on the Work of the Committee on Psychology—12 Years of Seminars on the Work of the Committee on Psychology”, there are 740,000 psychology committee members among undergraduate students and postgraduate students across all Chinese universities. In practice, this program is not identified as straightforward antistigma action: the responsibility of chosen committee members is simply to provide support, but this still results in reduced pressure from public discrimination. Similarly with the discussions raised by the existing research, at most universities, the role orientation and responsibilities of the psychology committee members are regarded as an essential problem for the development of psychology committee members. While they are always identified as volunteers or propagandists for the psychological activities, collectors for the psychological information from the students, these qualified members have a lack of activism and motivation in providing services for the peer classmates [33].

However, some complaints and concerns about negative influences from peers emerged from the discussions, and the influence of friends is also emphasized by the existing research. In our study, it is also clearly stated that some students are embarrassed to share with their friends their experiences of mental illness: they are afraid it may bring discrimination or prejudice. Moreover, some more serious influences are deployed from discourses made by the peers.

*“Some students have no clear awareness about themselves: they are easily influenced by the friends around them, and they choose to follow the others’ behavior. Some of my classmates made close relationships with the others, getting boyfriends or girlfriends only because their friends are not single anymore. I think this kind of “following” behavior would make horrible influences”.*

(2S1, p8, p5)

*“Once, one of my classmates talked to me, sharing his feelings of depression. To be honest, I can’t take this in my deep heart, even can’t accept this group of [mentally ill] people. I have my own life; I’m busy studying. It’s better if they don’t share their feelings with me”.*

(6S1, p11)

Peer influence or peer support was identified as a major factor for those Chinese students, embedded in the collective ideology of Chinese culture. Peer influence is not a theme that influenced the stigma of mental illness; however, in the study, its level of influence is still not fully understood. From the worldwide perspective, the idea of peer support is also a popular topic. The idea that people with mental health problems may receive support from others who share their experiences has a long history in mental health services. Some staff members are chosen from former mental patients, because they are better suited to help these persons: they have shared similar experiences with them and are usually more gentle, honest, and humane [34]. Aside from the themes presented, some factors that affect students’ mental health came to light from the statements of the focus groups. These included societal stressors such as a stigma towards mental illness and lack of mental health literacy; work and academic stressors such as competition for research and relationships with supervisors; relational stressors such as roommates, romantic relationships, and student clubs; family influences such as childhood experiences, growth environments, financial stressors; and so on.

## 5. Discussion and Implications

The discussed findings come from an inductive analysis of the focus groups of professional service providers and college students, and below we categorize the discussion and implications into the theoretical and practical domains.

With regard to the theoretical implications, from our empirical observations, especially via the focus group discussions, some subjective opinions enhanced our understanding of mental illness stigma among Chinese college students. Although subjective, individual-level experiences were discussed, and the social transitivity of mental illness stigma was still demonstrated. The stigma of mental illness is socially constructed, which reflects the perspective and responses to mental illness that people share. The stigma is regarded as a social problem rather than an individual problem and identified as a social process with transitivity [7,35]. During our research, we attempted to understand stigma as a complex social process that involves labeling, stereotyping, and peer influences rather than as a status of shame that an individual bears.

Moreover, mental illness stigma among college students is a worldwide problem, but this study explored the topic in Chinese culture. We argue that there could be different results if others were to place this study into the global research context, especially given the extreme peer influence shown in our focus groups (which, in this case, is related to Chinese collective ideology). According to Bedford and Hwang [9], in contrast to the individualism of Western culture, Chinese identity is always relational (i.e., in terms of a person’s system of relationships). Therefore, group-oriented behavior, such as harmonious interaction among group members, is highly valued. From this perspective, the danger from peers needs to be emphasized, and the support from them could make more contributions at the same time. In our study, positive peer influence inspired some respondents to decrease their stigmatizing of mental illness.

With regard to the practical implications, as discussed earlier, improving peer support is an innovative approach in reducing the stigma of mental illness, and training the students who would have a positive influence on their peers is one of the purposes of the ACE-LYNX program. Our study is part of the ACE-LYNX implementation study which “trains the trainers” with the ACE model (which integrates the ACT and Empowerment models). This program aims to decrease the stigma of mental illness, increase mental health knowledge, promote individual psychological wellbeing, promote the collective capacity to collaborate and promote mental health, and form an integrative network of care for Chinese college students. The program is to be conducted between 2018–2023 among six universities in Shandong, Jinan. Using the “train-the-trainer model”, 90 interdisciplinary professionals will be trained to become ACE-LYNX-Pros. They will then train the next generation of ACE-LYNX-Pro Champions (n = 270) and students to become ACE-LYNX Student Champions (n = 360), who will in turn provide support to Chinese college students or peers for mental health issues, using an applicable version of the intervention [26]. Other studies have concluded that targeting the general public through mass antistigma interventions may lead to a virtuous cycle that disrupts the negative feedback engendered by public stigma and thereby reduces self-stigma among people with mental health problems. A combined approach involving knowledge, attitudes, and behavior is needed. Mass interventions that facilitate disclosure and positive social contact may be the most effective means. Increasing the availability of information about mental health issues and facilitating access to care and help-seeking are also promising solutions to mental health stigma [36]. 

There are some limitations to the findings in this study that need to be considered in future research. Firstly, the qualitative data analysis for the focus group was more complex than that for the individual interviews. We adopted framework analysis as the data analysis strategy, and while this was still based on the purpose of thematic analysis, it is possible that more meaningful themes or findings were neglected because there were fewer techniques and experiences that could be used for the data analysis of the focus groups. Secondly, this study was conducted as the context survey for the ACE-LYNX program for promoting mental health among Chinese college students, so the questions listed within focus groups were descriptive questions rather than explanatory questions. The findings we presented were subjective and individual-level understandings of mental health and mental illness; however, there are still more deep questions focused on the issue of stigma that should be considered for future studies.

## 6. Conclusions 

As our findings show, the public stigma regarding mental illness includes a number of things: mental illness is regarded as severe, pathetic, and complicated, and for some parents and some students, even taking part in psychological counseling was considered a horrible existence. We also concluded in the process that stigma is internalized from the public to the individual, and also that peer influence was presented in both positive and negative ways. There are two main strengths of our work. Firstly, most mental health studies of college students in China conducted quantitative surveys to identify causal relationships; however, very limited studies have used qualitative methods to analyze mental illness stigma, and focus groups were conducted in data collection in our study to acquire a comprehensive understanding of the contexts of the participants’ insights and experiences. Secondly, this study is a part of the ACE-LYNX implementation study, which aims at mental illness stigma reduction among Chinese college students, and we attempt to construct the well-trained professionals and students who form an integrative network of care for Chinese college students. 

## Figures and Tables

**Table 1:** Top six barriers to treatment and support.

Barrier to Treatment and Support	Number of the Participants	Percentage of the Participants
1. Fear of judgement/discrimination from peers	450	75%
2. Fear of judgment/discrimination from the community	447	75%
3. Privacy and confidentiality concerns	438	73%
4. Lack of safe space/privacy for services	407	68%
5. Access barriers related to service locations/opening/hours/costs, etc.	384	64%
6. Lack of school/public policies to protect against mental illness discrimination	382	64%

## Data Availability

The original contributions presented in the study are included in the article; further inquiries can be directed to the corresponding author.
